# Development of a Fully Automated Flow Injection Analyzer Implementing Bioluminescent Biosensors for Water Toxicity Assessment

**DOI:** 10.3390/s100807089

**Published:** 2010-07-27

**Authors:** Efstratios Komaitis, Efstathios Vasiliou, Gerasimos Kremmydas, Dimitrios G. Georgakopoulos, Constantinos Georgiou

**Affiliations:** 1 Chemistry Laboratory, Agricultural University of Athens, 75 Iera Odos, 118 55 Athens, Greece; E-Mails: stratoskomaitis@yahoo.com (E.M.K.), egvasiliou@yahoo.com (E.G.V.); 2 Microbiology Laboratory, Agricultural University of Athens, 75 Iera Odos, 118 55 Athens, Greece; E-Mails: dgeorga@aua.gr (D.G.), gkrem@aua.gr (G.K.)

**Keywords:** *Vibrio fischeri*, toxicity, flow injection

## Abstract

This paper describes the development of an automated Flow Injection analyzer for water toxicity assessment. The analyzer is validated by assessing the toxicity of heavy metal (Pb^2+^, Hg^2+^ and Cu^2+^) solutions. One hundred μL of a *Vibrio fischeri* suspension are injected in a carrier solution containing different heavy metal concentrations. Biosensor cells are mixed with the toxic carrier solution in the mixing coil on the way to the detector. Response registered is % inhibition of biosensor bioluminescence due to heavy metal toxicity in comparison to that resulting by injecting the *Vibrio fischeri* suspension in deionised water. Carrier solutions of mercury showed higher toxicity than the other heavy metals, whereas all metals show concentration related levels of toxicity. The biosensor’s response to carrier solutions of different pHs was tested. *Vibrio fischeri’s* bioluminescence is promoted in the pH 5–10 range. Experiments indicate that the whole cell biosensor, as applied in the automated fluidic system, responds to various toxic solutions.

## 1. Introduction

Current legislation in the EU (European Groundwater Directive) requires that water quality and the degree of contamination be assessed using chemical methods. Such methods do not consider the possible synergistic or antagonistic interactions that may affect the bioavailability and toxicity of pollutants in the environment [1]. Bioassays are methods for assessing the toxic impact of whole samples on the environment and for screening environmental samples before going onto detailed chemical analyses that can be time consuming, expensive and do not allow monitoring [2]. The utilization of organisms possessing lux—genes [3] gained significant importance during the last decade since toxicity bioassays have been recognized as essential tests along chemical analyses [4]. The widely used marine photobacterium *Vibrio fischeri* is a self—maintained luminescent unit. The level of *in vivo* luminescence reflects the metabolic rate of luminous bacteria and the integrity of the bacterial cells [5].

In recent years, the development of whole-cell biosensors has seen increasing interest due to the capability of whole cells to convert complex substrates using specific metabolic pathways [6] and because of potential applications of whole-cell biosensors for monitoring of typical *sum parameters*. Such parameters, such as toxicity [7], biological oxygen demand [8] xenobiotic compounds [9] or heavy metals [10] cannot be monitored using enzyme-based sensors. The opportunity to modulate metabolic activities of specific cells can additionally be used for drug screening [11] and combinatorial approaches for drug discovery [12]. Additionally, microbial biosensors have been successfully applied for the specific determination of single components such as e.g., glucose, fructose, xylose and alcohols [13]. A general advantage of microbial biosensors is that living cells are continuously repairing their integrated enzyme activities and enzyme cascades. This is a clear advantage in comparison to biosensors based on labile biological recognition elements (e.g., enzymes). On the other hand, the development of such biosensors allows the use of immobilized organisms that maintain their physiological status and thus the results obtained will represent the natural responses.

A further search of the literature shows that few articles describe the implementation of these devises in automated analyzers for the construction of integrated analytical instrumentation for real sample analysis and monitoring [14].

Flow Injection (FI) is a mature technique. Recent examples are the determination of total antioxidant capacity of virgin olive oil and the multidetermination of heavy metals as described in references [15] and [16]. Flow techniques offer many advantages in the automation of wet chemistry methods while they can not readily automate analysis of solid materials. This article describes the development of a fully automated Flow Injection analyzer for the implementation of bioluminescent biosensors in toxicity assessments. The function and use of the developed system are assessed through the luminol peroxidase system and the response to toxic heavy metals using *V. fischeri* bacteria.

## 2. Experimental Section

### 2.1. *V. fischeri* Culture

*V. fischeri* strain NRRL-11177 (Dr Lange S.A.) was grown in 20 mL DSMZ No 6904 broth [4] for 20 h in an orbital incubator at 190 rpm at 24 °C [3]. The thus developed stock culture was mixed with 30% glycerin, dispensed in 2 mL vials and stored at −70 °C. Twenty μL of the stock culture were inoculated in 50 mL of growth medium and grown overnight. Then the culture was centrifuged and resuspended in artificial sea water, preserving their function but stopping their growth. This suspension was used with the FI system.

### 2.2. Chemicals

All chemicals used in the system were of analytical reagent grade purchased from Merck (Darmstadt, Germany). 0.0200 M Hg^2+^, Cu^2+^ and Pb^2+^ stock solutions were prepared by dissolving HgCl_2_, Cu(SO_4_)·5H_2_O, Pb(NO_3_)_2_·H_2_O. Acetate and phosphate buffer solutions were prepared for the pH ranges 3–6 and 7–11, respectively. pH was adjusted with NaOH and HCl.

## 3. Results and Discussion

### 3.1. Detector Unit Development

Light detection device: Photomultipliers (PMTs) are the most sensitive devices for the detection of low level optical signals. An integrated PMT tube, incorporating the high voltage source and divider circuit along with an RS 232 signal output was selected (Hamamatsu HC-135 01) resulting in a compact biosensor device.

**Figure 1.**
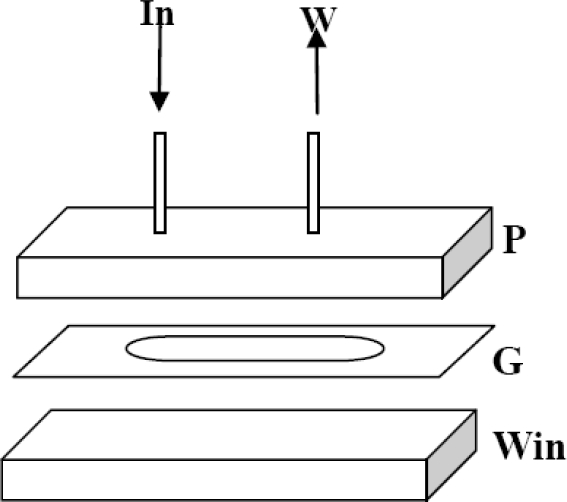
Flow cell design. **In:** carrier solution feed; **W:** waste; **P:** Plexiglas plate with immobilized cells; **G:** Gasket, 2mm thickness, 0.8cm^2^ area; **Win:** optical window from Plexiglas or Quartz (PMT detector is opposite). Dimensions after assembly: 2 cm × 1 cm × 1 cm. Internal volume of flow cell is 160 μL.

Flow cell: Due to the use of a PMT detector, a wall-jet flow cell configuration was implemented. Figure 1 depicts the design of the prototype based on a previous study by Divritsioti *et al.* [7]. The assembly was clamped between two stainless steel plates featuring appropriate openings for light detection and passage of the **In** (entrance of carrier solution) and **W** (exit of carrier solution to the wastes) polytrafluoroethylene (PTFE) tubes of 0.8 mm internal diameter.

Temperature control of the flow cell: To maintain immobilized bacteria at their optimal temperature, the flow cell is enclosed in a thermostated aluminium frame using a water bath at 20 °C.

### 3.2. Flow Injection Analyzer Development and Optimisation

**Figure 3.**
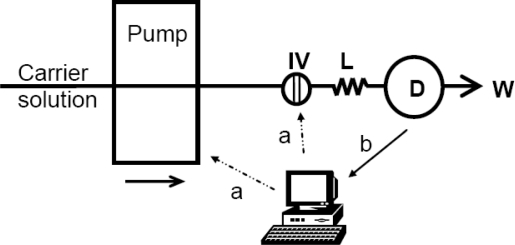
Automated Flow Injection Analyzer. **D:** detector unit; **IV:** injection valve; **L:** mixing coil; **W:** waste; a: digital control signals; and b: data acquisition line.

The single line Flow Injection system that was developed, incorporating the above described detector unit, is depicted in Figure 3.

The analyzer design is based on the continuous flow of a carrier solution. The sample loop of the injection valve is loaded with the *Vibrio fischeri* cell suspension that is subsequently automatically injected in the carrier solution flow. *Vibrio fischeri* cells are mixed with the carrier solution in the mixing coil and then driven to the detector unit for bioluminescence assessment. % Inhibition of bioluminescence is assessed with a two step experimental protocol. The first step is the assessment of *V. fischeri* bioluminescence by injection in a non toxic carrier solution. The second step is the assessment of *V. fischeri* bioluminescence by injection in a toxic carrier solution (sample). %Bioluminescence inhibition is calculated using the readouts (peaks) from the two injections: %Bioluminescence inhibition = (Peak 1–Peak 2) × 100/Peak 1

The thus calculated bioluminescence inhibition correlates with the toxic effect of the sample to *V. fischeri* cells. For the evaluation of the peak readouts, both heights and areas have been assessed. *V. fischeri* wash out from the analyzer was a slow procedure, resulting in extensive peak tailing. This was probably due to the shape of the flow cell that does not facilitate the wash out of bacteria. To overcome this problem and shorten the analysis time, 2 s after recording the peak maximum, the analyzer pump was set to maximum flow rate (∼10 mL/min). In this way, the turnover time for a single peak was kept to just 40 s. Precision of peak height measurements assessed through 10 injections of the same *V. fischeri* culture was found to be 0.7% RSD. Peak area measurements resulted in lower precision presumably due to the high flow rate used for the wash out step.

Three different volumes of *V. fischeri* suspension were tried: 80, 100 and 200 μL. Signal increased with increasing the injected volume. Although the signal is maximized when injecting 200 μL of *V. fischeri* culture, we choose 100 μL in order to minimize consumption of the culture. In this way, 12 injections were feasible using the same culture suspension. Although just 100 μL are injected, another portion of around 400 μL is consumed while filling the sample loop of the injection valve. This is actually a general disadvantage of the procedure of filling a sample loop through aspiration. Although this disadvantage could be overcome, when using conventional reagents, by replacing aspiration through the use of a syringe, this is not an option when using bacterial suspensions as reagents. Bacterial membranes could be partialy disrupted resulting in loss of signal. When using 8 mL culture suspension 16 injections are possible. It should be noted that *V. fischeri* culture cannot be used the way reagents are used: upon standing, due to gravity, bacteria tend to accumulate in the bottom of the container. This results in a gradual signal decrease that was up to 30% for one hour operation and diminished precision. To overcome this problem, a small volume culture (8 mL) that was continuously stirred with a magnetic stirrer, was used.

Flow rate was optimized in the range of 0.5 to 2.0 mL/min. At flow rates lower than 1.5 mL/min peaks were not sharp and the whole procedure was slow resulting in high turnover time. Flow rates higher than 1.5 mL/min resulted in diminished precision (RSD’s up to 5%). The flow rate of 1.5 mL/min selected as a compromise between fast washout of *V. fischeri* cells from the analyzer and precision. Mixing coils of 50, 100 and 150 cm have been tested. The 100 cm mixing coil was selected as a compromise between adequate mixing and analysis time.

### 3.3. Software

**Figure 4.**
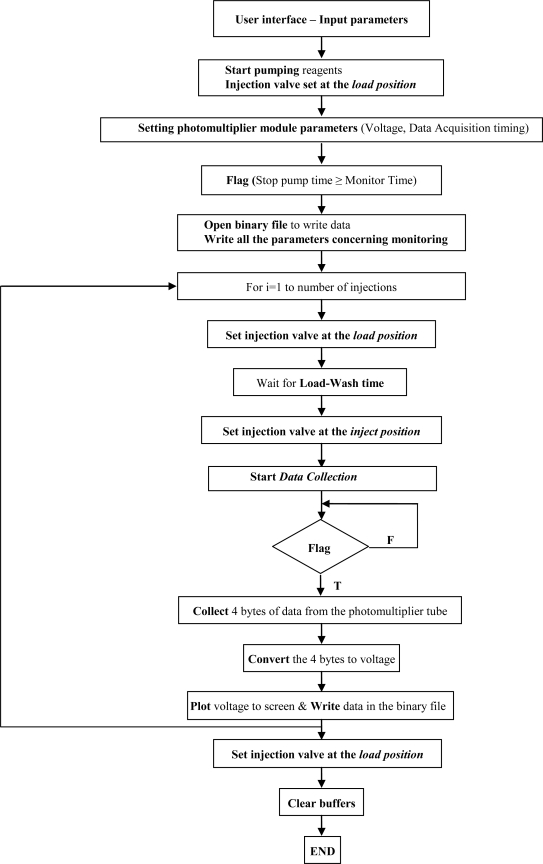
Data acquisition and control program flow chart. **F**: false; **T**: true.

The software package developed in Labview language provides functions for data acquisition from the biosensor unit, control of the peristaltic pump and the injection valve, bubble detection and subsequent correction of FI peaks, quantitative measurements, drifting baseline correction and export of data to text files. The data acquisition and control program flowchart is depicted in Figure 4:

### 3.4. Assessment of Heavy Metals Toxicity

**Figure 5.**
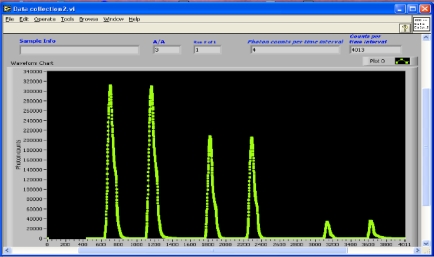
Typical FI toxicity assessment diagram. Horizontal axis: time (s), Vertical axis: Bioluminescence Intensity (PMT counts). Peaks from left to right: blank (1st and 2nd), Cu^2+^ solution 3.0 mM (3rd and 4th) and 10.0 mM (5th and 6th).

**Figure 6.**
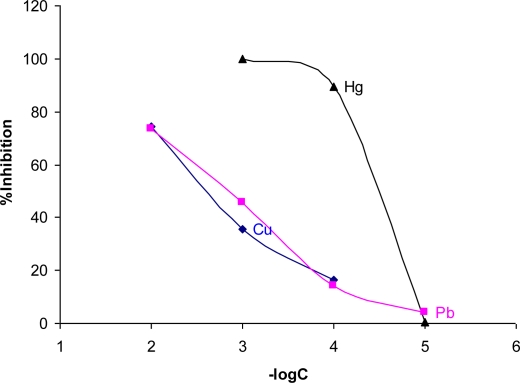
Bioluminescence inhibition dose-response curves for Hg^2+^, Pb^2+^ and Cu^2+^. It is evident that Hg^2+^ shows the highest toxicity when tested with the developed system.

The developed Flow Injection system was used for the assessment of toxicity due to different heavy metals. A typical FI toxicity assessment output is shown in Figure 5, while the dose response curves for Cu^2+^, Pb^2+^ and Hg^2+^ are depicted in Figure 6. It is clear that the developed system is able to assess toxicity due to heavy metals present in water samples. Effective concentrations were in the range of 1.0 × 10^−2^ M–1.0 × 10^−5^ M.

It should be noted that the low limit of the useful concentration range is not much lower than that presented in Figure 6. The work presented here is showing the principle of using cells as a “reagent” in flow systems in order to use the system in water analysis. Further work is needed to lower the detection limits. If needed detection limits can be modulated by stopping the flow – after stopping the flow for 15 minutes detection can go down to 10^−7^ M, depending on the toxic metal compound.

**Figure 7.**
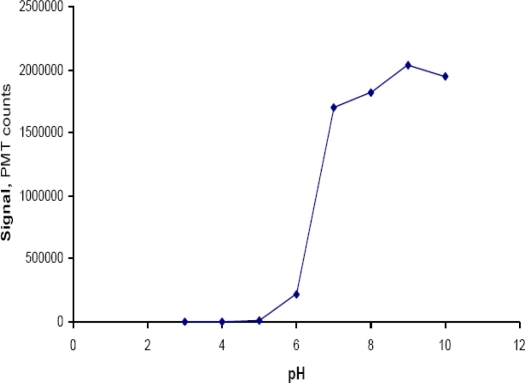
Signal resulting from a *V. fischeri* culture injected in carrier solutions of different pHs.

Hydrolysis of metal salts modulates the pH of aqueous solutions. To assure that the systems’ response was due to heavy metal content and not to pH changes during dissolution of metal salts, we checked the response to carrier solutions of different pHs in the range of 3–10 using as blank distilled water (pH = 5.50). Results depicted in Figure 7 show that *V. fischeri’s* bioluminescence increases along the pH range from pH 5 to pH 10. It must be noted that the pH of the suspension of *V. fischeri* cells of in the reconstitution solution (pH 5.21) was 6.22.

The pH range of 1.0 × 10^−2^–1.0 × 10^−5^ M Hg^2+^ was 3.75–4.47. For 1.0 × 10^−2^–1.0 × 10^−4^ M Cu^2+^ and Pb^2+^ pH was in the range of 4.24–4.93 and 4.23–5.00, respectively. According to Figure 7 the signal is this pH range is completely lost. At this point it should be noted that results presented in Figure 7 refer to solutions of phosphate and acetate buffers prepared as described in the reagents section. However, as shown in Figure 6, when using metal solutions inhibition is differentiated.

To measure dispersion we first recorded the signal (D_s_) of the bioluminescent suspension using it as a carrier stream and then the signal from 100 μL the same suspension injected in a carrier stream of deionised water (D_o_). The D_s_ signal divided by D_o_ gives the dispersion which in the developed FIA system was calculated and found to be equal to 4.52. That means that the pH in the flow cell is between 5.50 (pH of deionised water) and 6.22 (pH of the reconstituted suspension of *V. fischeri* cells).

## 4. Conclusions

This paper shows the development of an automated Flow Injection analyzer for water toxicity assessment based on *V. fischeri* bioluminescent bacteria. It is an initial work that we plan to expand to various toxic compounds and real samples in the near future. This microbial biosensor proved to be able to measure toxicity of three heavy metals based on their inhibition of *V. fischeri* bioluminescence. The biosensor was able to measure inhibition rapidly with precision of 0.7% RSD. The lower limit of the calibration curves were 1.0 × 10^−4^ M for Pb^2+^, 1.0 × 10^−4^ M Cu^2+^, and 1.0 × 10^−5^ M for Hg^2+^.

## Figures and Tables

**Figure 2.**
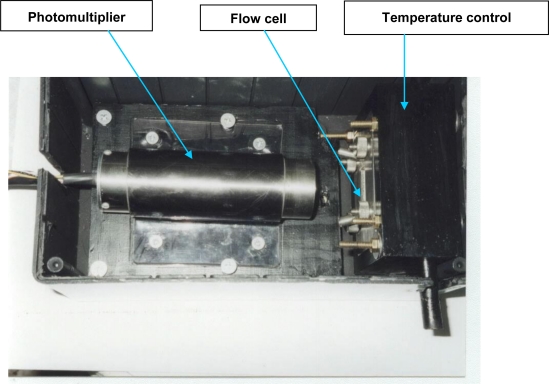
Detector unit incorporating photomultiplier tube, flow cell and temperature control of the flow cell.
